# Delayed Infection of Tissue Expanders in Immediate Two-Stage Implant-Based Breast Reconstruction

**DOI:** 10.1055/a-2822-5229

**Published:** 2026-03-27

**Authors:** Hye Ju Han, Ahran Kim, Min Suk Park, Daiwon Jun, Jiyoung Rhu, Pill Sun Paik, Jung Ho Lee

**Affiliations:** 1 Department of Plastic and Reconstructive Surgery, Bucheon St. Mary's Hospital, College of Medicine, The Catholic University of Korea, Seoul, Republic of Korea; 2 Department of Surgery, Bucheon St. Mary's Hospital, College of Medicine, The Catholic University of Korea, Seoul, Republic of Korea

**Keywords:** implant-based breast reconstruction, risk factors, surgical wound infection, tissue expansion devices

## Abstract

**Background:**

While surgical site infection (SSI) is a major complication of implant-based breast reconstruction, delayed infections remain underrecognized despite their clinical significance. This study aimed to identify the risk factors of delayed infection and compare the clinical outcomes of acute and delayed SSI after tissue expander insertion.

**Methods:**

Patients who underwent immediate tissue expander-based breast reconstruction between March 2016 and February 2021 were reviewed. Acute SSI (<30 days) and delayed SSI (>60 days) were analyzed and compared with the no-infection group.

**Results:**

Among 146 breasts (140 patients), 26 SSIs occurred; 50% ( *n* = 13) were delayed. Multivariable analysis identified wound complication as the sole independent risk factor for delayed SSI. Compared with acute SSI, delayed infection was associated with a significantly longer interval from symptom onset to diagnosis (4.15 vs. 0.33 days, *p* = 0.0181) and a lower salvage rate (31% vs. 83%), showing borderline significance ( *p* = 0.057).

**Conclusion:**

Delayed SSI is not rare after expander-based breast reconstruction. Because salvage is challenging due to diagnostic delay, surgeons and patients should remain vigilant, particularly when risk factors for delayed infection of the tissue expander remain.

## Introduction

Breast reconstruction using an implant is becoming increasingly common due to its simplicity and the avoidance of donor site morbidity. 1 2 3 However, complications—including seroma, hematoma, capsular contracture, and infection—may develop following implant-based breast reconstruction, and infection is among the most serious causes of reconstruction failure. Especially, when surgical site infection (SSI) develops, long-term antibiotic treatment, hospital readmission, or implant removal may be required for controlling SSI. 4

The reported infection rate following implant-based breast reconstruction ranges from 1% to 35%. This broad variation may be attributed to differences in the definition of infection and the retrospective nature of many studies with variable follow-up durations. In implant-based breast reconstruction, SSI usually refers to infection occurring within 30 days postoperatively. 5 6 However, late complications also occur. Some studies define infections occurring between 30 and 60 days as “subacute” and those occurring beyond 60 days as “delayed.” 3 7

Delayed infection is particularly important because it often develops insidiously, is more difficult to diagnose, and is less likely to be salvaged once established. Nevertheless, most studies to date have primarily focused on acute infections, leaving the clinical features and risk factors for delayed infection insufficiently characterized. In particular, limited attention has been paid to postoperative wound complications as a determinant of delayed SSI during prolonged tissue expander retention.

Therefore, this study aimed to (1) identify risk factors associated with delayed (>60 days) SSI after tissue expander insertion, and (2) compare clinical outcomes between acute and delayed infections in immediate two-stage implant-based breast reconstruction.

## Methods

From March 2016 to February 2021, patients with breast cancer who underwent immediate breast reconstruction using a tissue expander at a single institution were included for a retrospective medical record review. As this study was retrospective in nature, a formal a priori power analysis was not performed. Instead, all consecutive patients who met the predefined inclusion criteria during the study period were included to maximize the available sample size. Exclusion criteria included (1) direct-to-implant reconstruction (due to fundamentally different perioperative course and infection dynamics), (2) delayed or autologous reconstruction, (3) follow-up shorter than 12 months, and (4) TE removal for reasons other than infection. Infections that developed after the second-stage permanent implant placement were excluded.

All patients underwent breast reconstruction using a tissue expander (Mentor® CPX™ 4; Mentor Corp., Santa Barbara, CA, style 9200) ranging from 275 to 550 mL in volume. In all cases, a fusiform incision encompassing the nipple–areolar complex and the tumor lesion was used. Intraoperative assessment of mastectomy flap vascularity was based on clinical judgment, including flap color, capillary refill, and bleeding at the skin edge. During hospitalization, cefazolin was used for prophylaxis of infection. The drain was removed when the output was <30 mL per day for 2 consecutive days. Expander inflation was started 2 weeks postoperatively on an outpatient basis.

SSI was defined according to the Centers for Disease Control and Prevention (CDC) definition of a deep SSI, ensuring consistency with international standards. It is characterized by three or more of the following findings: Pain, local swelling, erythema, pus, fever, seroma, wound dehiscence, and skin perforation. 8 We classified SSI as acute (<30 days), subacute (30–60 days), and delayed (>60 days) after surgery, 7 to allow comparison across infection timing groups.

To identify the risk factors for delayed SSIs, data on preoperative, intraoperative, and postoperative factors were gathered. Preoperative factors included age, body mass index (BMI), diabetes mellitus, hypertension, and tobacco smoking. Intraoperative factors encompassed operation time, mastectomy type, axillary lymph node dissection, implant insertion plane (prepectoral or subpectoral), with all subpectoral reconstructions performed using a dual-plane technique with acellular dermal matrix coverage of the lower pole, use of acellular dermal matrix (MegaDerm®, L&C Bio Inc., Seongnam, South Korea), mastectomy specimen weight, and initial tissue expander inflation volume. Mastectomy types included skin-sparing and nipple-sparing mastectomy. Total tissue expander inflation volume, total drainage volume, and drainage duration, including the total amount until the drain was removed and the amount of seroma aspirated, were the postoperative variables. Additionally, adjuvant radiotherapy, chemotherapy, and postoperative complications were included for evaluation. Given the limited number of delayed infection events, further stratification of chemotherapy timing was not performed to avoid sparse data bias and model instability.

Wound complications included postoperative seroma and skin necrosis. Seroma was defined as a clinically or radiologically confirmed fluid collection requiring aspiration or prolonged drainage, whereas skin necrosis was defined as partial- or full-thickness mastectomy flap necrosis requiring conservative or surgical management.

Given the limited sample size and the small number of delayed SSI events, these complications were analyzed as a composite variable to ensure statistical stability and to reflect postoperative wound conditions potentially associated with impaired tissue integrity during the tissue expansion period.

The no-infection group was used as a reference for identifying potential risk factors, and univariable and multivariable analyses were performed. Delayed SSI was analyzed as a binary outcome rather than as a time-to-event variable because the number of delayed infection events was limited. Therefore, logistic regression analysis was considered more appropriate than a Cox proportional hazards model for identifying associated risk factors. We reported odds ratios, 95% confidence intervals, and corresponding *p* -values on the basis of the model. Statistical analyses were performed using GraphPad Prism version 9.0.1 (GraphPad Software Inc., San Diego, CA), and a two-sided *p* < 0.05 was considered statistically significant. Given the limited number of delayed SSI events, multivariable analyses were considered exploratory and were interpreted with caution. Model discrimination was assessed using the area under the receiver operating characteristic curve, and calibration was evaluated using the Hosmer–Lemeshow goodness-of-fit test, with cautious interpretation given the small sample size.

Additionally, the clinical characteristics and prognosis between the acute and delayed infection groups were compared. The grades of postoperative implant infection were defined on the basis of a previous study as follows: Grade 1 (possible), including fever, minimal local edema, and inflammation resolution with empiric antibiotic therapy; grade 2 (probable), including cellulitis, leukocytosis, systemic inflammation, echographic evidence of inflammation, or periprosthetic liquid accumulation, without microorganism isolation from echographic needle aspiration or blood culture; and grade 3 (proven), including criteria for probable infection with cellulitis, presence of purulent discharge, and microorganism isolation. 7

## Results

### Demographic Data

Overall, 161 breasts (154 patients) that underwent immediate breast reconstruction using a tissue expander from March 2016 to February 2021 following breast cancer diagnosis and undergoing mastectomy were included in this study. Of these, 15 breasts (14 patients) were excluded after applying the exclusion criteria. SSI occurred in 26 breasts (17.8%). Among these, 6 (23.1%) were acute (<30 days), 7 (26.9%) were subacute (30–60 days), and 13 (50.0%) were delayed (>60 days). Table 1 presents baseline demographic characteristics and univariable analyses comparing the no-infection and delayed SSI groups. The mean follow-up period was 211.51 days. In cases wherein delayed infection developed, the time point of infection was considered the follow-up endpoint (173.15 days).

**Table 1:** Comparison of demographic characteristics and univariable analysis of potential risk factors for delayed surgical site infection

	No infection	Delayed infection	*p* -Value
*N*	120	13	–
Onset of infection after surgery		173.2	–
Follow-up (days)	211.51	173.15	–
Age (years)	51.07	49.54	0.5074
BMI (kg/m ^2^ )	21.41	24.9	0.0234 a
DM	8 (6.67%)	1 (7.69%)	>0.9999
HTN	14 (11.67%)	3 (23.08%)	0.3731
Smoking	13 (10.83%)	4 (30.77%)	0.0636
Operation time (minutes)	253.1	273.8	0.2586
ALND	27 (22.50%)	8 (61.54%)	0.0053 b
Plane			
Prepectoral	37 (30.83%)	7 (53.85%)	–
Subpectoral	83 (69.17%)	6 (46.15%)	0.1219
ADM	110 (91.67%)	11 (84.62%)	0.3318
Mastectomy weight (g)	386.9	492.3	0.0455 a
Total inflation volume (mL)	359	330.4	0.3458
Initial inflation (mL)	71.13	68.46	0.8806
Postop inflation (mL)	287.9	261.9	0.3800
Total drainage volume (mL)	772.8	1259	0.0004 c
Drain duration (days)	10.34	12.46	0.0649
Radiotherapy	30 (25.00%)	9 (69.23%)	0.0021 b
Chemotherapy	78 (65.00%)	12 (92.31%)	0.0602
Neoadjuvant	3 (2.5%)	0	–
Adjuvant	66 (55%)	6 (46.15%)	
Combined	9 (7.5%)	6 (46.15%)	
Wound complication (seroma or skin necrosis)	45 (37.50%)	11 (84.62%)	0.0019 b

Abbreviations: ADM, acellular dermal matrix; ALND, axillary lymph node dissection; BMI, body mass index; DM, diabetes mellitus; HTN, hypertension.

a *p* < 0.05 using Student's *t* -test and Fisher's exact test.

b *p* < 0.01 using Student's *t* -test and Fisher's exact test.

c *p* < 0.001 using Student's *t* -test and Fisher's exact test.

### Delayed Surgical Site Infection

Univariable analyses revealed that the delayed SSI group (24.9 kg/m ^2^ ) had a significantly higher BMI than the no-infection group (21.4 kg/m ^2^ ; *p <* 0.05). Furthermore, the delayed SSI group had significantly higher ALND rates ( *p* < 0.01). The delayed SSI group (492.3 g) had a significantly greater mastectomy specimen weight than the no-infection group (386.9 g; *p* < 0.05). Of the postoperative factors, the delayed infection group (1,259 mL) had a significantly higher total drainage volume than the no-infection group (772.8 mL; *p* < 0.01). Furthermore, radiotherapy (69.23%; *p* < 0.01) and complication (84.62%; *p* < 0.01) rates were significantly higher in the delayed infection group than those in the no-infection group ( Table 1 ).

Multivariable analyses revealed that wound complication was an independent risk factor for delayed SSI (odds ratio, 7.020; 95% confidence interval, 1.313–58.48; *p* = 0.03; Table 2 ).

**Table 2:** Multivariable analysis of potential risk factors for delayed surgical site infection

Variable	OR	95% CI	*p* -Value
BMI (kg/m ^2^ )	1.205	0.9785–1.529	0.0923
Smoking	5.624	0.8376–41.28	0.0739
ALND	9.805	0.1720–721.3	0.2768
Mastectomy weight (g)	0.9986	0.9926–1.004	0.6040
Total drainage volume (mL)	1.001	0.9990–1.002	0.4636
Radiotherapy	3.599	0.3360–41.18	0.2836
Chemotherapy	7.032	0.5926–238.7	0.1813
Wound complication	7.020	1.313–58.48	0.0362 a

Abbreviations: ALND, axillary lymph node dissection; BMI, body mass index; CI, confidence interval; OR, odds ratio.

a *p* < 0.05 using multivariable logistic regression.

*p* < 0.01 using multivariable logistic regression.

### Clinical Characteristics of Delayed Surgical Site Infection

To characterize the clinical course of the delayed SSI group, it was compared with the acute SSI group ( Table 3 ). Localized symptoms and signs of infection, including swelling, heating sensation, pain, and tenderness, appeared at an average of 18.17 ± 6.49 and 173.15 ± 112.73 days postoperatively in the acute and delayed SSI groups, respectively. No significant differences were observed in the infection grade, presence of systemic and localized symptoms, fever, hospitalization period, or isolation of delayed bacteria from tissue cultures between the two groups. However, a significant difference was noted in the time from initial symptom onset to diagnosis, with 0.33 and 4.15 days for acute and delayed SSIs, respectively. Although the delayed SSI group had higher leukocytosis, white blood cell (WBC) count, C-reactive protein (CRP) level, and erythrocyte sedimentation rate (ESR), the differences were not statistically significant. Of the six breasts diagnosed with acute SSI, two were successfully salvaged with their tissue expanders using intravenous antibiotics alone. Three breasts were salvaged by replacing the tissue expanders, whereas one breast required complete removal along with the tissue expander. Of the 13 breasts diagnosed with delayed SSI, surgical intervention (removal of the original expanders) was necessary for all cases, four of which were salvaged using new tissue expanders. Owing to the severity of infection, nine breasts could not be salvaged, two of which underwent delayed breast reconstruction using deep inferior epigastric perforator (DIEP) flaps.

**Table 3:** Analysis of clinical features between acute and delayed infection

	Acute infection	Delayed infection	*p* -Value
*N*	6	13	–
Onset of infection after surgery	18.17	173.2	0.0041 a
Onset of infection after last radiation		71.12	–
Onset of infection after last expansion		119.88	–
Infection grade			
1	0	0	–
2	2 (33.33)	6 (46.15%)	>0.9999
3	4 (66.67%)	7 (53.85%)	–
Systemic symptom/sign	5 (83.33%)	9 (69.23%)	>0.9999
Peak body temperature (°C)	37.55	37.88	0.4879
Localized symptom/sign	5 (83.33%)	13 (100%)	0.3158
Diagnosis interval (day) ^c^	0.3333	4.154	0.0181 b
Hospitalization period (day)	12.50	10.23	0.6572
Isolated bacteria from tissue culture	4 (66.67%)	7 (53.85%)	>0.9999
MSSA	0	2	–
MRSA	1	0	
MRCNS	2	2	
*Streptococcus agalactiae*	0	1	
*Enterococcus faecalis*	0	1	
*Pseudomonas aeruginosa*	0	1	
*Enterobacter aerogenes*	1	0	
No growth	2 (33.33%)	6 (46.15%)	
Leukocytosis d	1 (16.67%)	8 (61.54%)	0.1409
WBC (10 ^9^ /L)	7782	15906	0.0540
CRP level (mg/L)	49.37	113.2	0.0951
ESR level (mm/h)	49.00	67.75	0.0911
Prognosis			
Salvage	5 (83%)	4 (31%)	0.0573
With only antibiotics	2	0	–
With new tissue expander	3	4	
Tissue expander removal	1	9	

Abbreviations: CRP, C-reactive protein; ESR, erythrocyte sedimentation rate; MRCNS, methicillin-resistant coagulase-negative *Staphylococcus* ; MRSA, methicillin-resistant *Staphylococcus aureus* ; MSSA, methicillin-sensitive *Staphylococcus aureus* ; WBC, white blood cell.

a *p* < 0.01 using Student's *t* -test and Fisher's exact test.

b *p* < 0.05 using Student's *t* -test and Fisher's exact test.

c Diagnosis interval (days): Time from the onset of symptoms to diagnosis of infection.

d Leukocytosis: Elevated WBC count above 11.0 × 10 ^9^ /L on a peripheral blood smear.

**Fig. 1:**
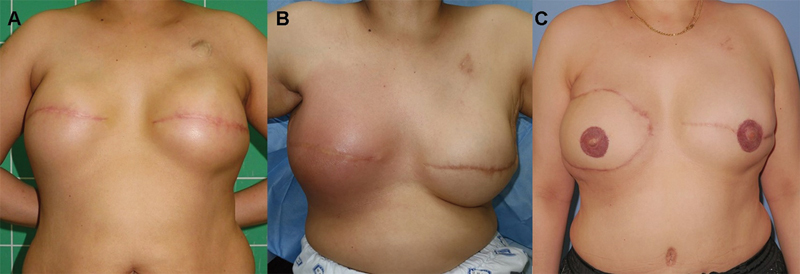
Clinical case of a 43-year-old patient who developed delayed SSI following immediate breast reconstruction using a tissue expander. ( **A** ) Immediately following the completion of tissue expander inflation. ( **B** ) The patient visited the emergency department with signs of infection, 487 days after the prior surgery. ( **C** ) One year following breast reconstruction was performed using a DIEP flap. DIEP, deep inferior epigastric perforator; SSI, surgical site infection.

A clinical case of a patient who developed delayed SSI following immediate breast reconstruction using a tissue expander is shown in Fig. 1 . The 43-year-old patient underwent a modified radical mastectomy for invasive ductal cancer of the right breast. She also had ductal carcinoma in situ in the left breast and underwent a simple mastectomy. After mastectomies, immediate bilateral breast reconstruction using tissue expanders and acellular dermal matrix (MegaDerm®, L&C Bio Inc., Seongnam, South Korea) was performed. Chemotherapy (four cycles of Adriamycin and cyclophosphamide followed by four cycles of docetaxel) and radiotherapy to the right chest wall and axilla (5,040 cGy/28 fx) were performed postoperatively; 487 days after the surgery and 383 days after the last expansion, the patient experienced a sudden onset of fever and right breast swelling, respectively. Three days following symptom onset, the patient visited the hospital with signs of infection, including high fever (39.8 °C), erythema, swelling, and warmth on the right breast. A WBC count of 12.190 × 10 ^9^ /L confirmed leukocytosis, and elevated levels of inflammatory markers, such as CRP (249.8 mg/L) and ESR (59 mm/h), were observed. The infection did not improve despite the use of intravenous antibiotics, leading to the removal of the expander and acellular dermal matrix. One month following explantation, secondary breast reconstruction was performed using a DIEP flap and contralateral insertion of an implant. No complications developed during the 1-year follow-up period.

## Discussion

When performing implant-based breast reconstruction, SSI usually refers to an infection that occurs within 30 days after surgery. 5 6 However, delayed infection may also occur after implant surgery, possibly due to bacterial colonization or biofilm formation on the expander surface. For spine implants, a 1% to 6.9% incidence of delayed infection has been reported, 9 10 11 12 and for hip and knee joint implants, delayed infection occurs in fewer than 1% of patients. 13 14

Previous experimental studies have demonstrated that tissue expanders may be permeable to bacteria, serving as potential bacterial reservoirs. 15 In addition, even saline-filled implants have been shown to harbor viable microorganisms within or on their surfaces, 16 suggesting that implant-related infections can develop long after the initial surgery.

Despite these findings, only a few studies have addressed delayed infections in implant-based breast reconstruction, with reported rates ranging from 33% to 71%. 3 7 17 Moreover, most studies on implant infection after breast reconstruction have focused on the first 60 postoperative days, and few have specifically examined delayed infections involving tissue expanders themselves, as they often did not differentiate between expanders and permanent implants. 3 6

In two-stage breast reconstruction, the period between the first and second operations can be extended due to adjuvant therapies. A recent review of tissue expander-based breast reconstruction reported that the average interval to second-stage implant placement ranges from 6 to 12 months, depending on adjuvant therapy and patient condition, including chemotherapy regimens that may require up to 12 cycles. 18 19 20 Furthermore, after radiotherapy, the interval is more prolonged (at least 4–5 months after the completion of radiotherapy) due to the risk of wound complications. 21 22 Consequently, it becomes crucial to conduct studies specifically focused on delayed infections related to expanders, and both patients and doctors should be aware of this potential outcome.

In our cohort of 161 breasts, infection developed in 26 breasts (17.8%), and 50% of these were delayed. This finding is consistent with the reported high incidence of delayed infection after implant-based breast reconstruction. In univariable analyses, our study showed the risk factors for delayed infection encompassed higher BMI, mastectomy weight, ALND, total drainage amount, radiotherapy, and wound complications. In addition, multivariable analyses showed a significant correlation with wound complications for delayed infection.

Higher BMI, increased mastectomy weight, and larger expander size (>400 mL) have been recognized risk factors for SSI. 3 23 24 25 26 These factors likely reflect longer operation time, larger flap surface area at risk for necrosis, and difficulties in detecting residual seroma in obese patients.

Following ALND, the likelihood of implant infection is sixfold higher than when no lymph nodes are removed. This finding can be explained by changes in the local immune response due to local lymphatic disruption. 27 Moreover, lymph node dissection potentially increases the risk of lymphatic leakage into the wound pocket, thereby increasing drainage duration, total postoperative drainage volume, and seroma formation. 28 Thus, patients who undergo ALND are more likely to experience unnoticed seroma collection, with a higher risk of delayed SSI.

Drain use duration and total drainage volume may also increase the infection risk. Chen et al 29 reported that drain duration was a significant infection risk factor for prosthesis-based breast reconstruction, and the odds ratio of infection increased by 76.2% with every additional week of drain duration. Surgical pocket healing is hindered by fluid accumulation, and a large amount of seroma reduces flap circulation by the distention of the skin flap above the tissue expander, thereby resulting in wound dehiscence and marginal necrosis. 30

Francis et al 25 reported that infection developed in 68 of 413 cases involving tissue expanders for postmastectomy breast reconstruction, with increased infection rates in irradiated implants ranging from 14% to 25%. Similarly, Nahabedian et al 21 indicated that among 168 breast reconstructions using expanders and implants, those that received radiation therapy had an infection rate that increased from 4.1% to 17.4%. These findings are because radiation therapy induces soft tissue fibrosis and injury to the microvasculature, which are impediments to normal wound healing and can cause minimal residual cutaneous damage, thereby resulting in barrier breakage and enabling bacterial seeding. 31 32

In addition to radiotherapy, adjuvant chemotherapy has also been reported to increase the risk of postoperative complications and infection by impairing host immune response and delaying wound healing. 33 Therefore, the combined effects of chemotherapy and radiation may further predispose patients to delayed infection following expander-based reconstruction.

Finally, wound complications may be associated with the development of delayed SSIs. Although seroma and skin necrosis represent distinct clinical entities, they were analyzed as a composite wound complication variable due to the limited number of events. This approach was intended to improve statistical robustness rather than to suggest equivalent clinical impact or mechanisms.

Because wound complications were the only factor independently associated with delayed SSI, preventive strategies aimed at optimizing wound healing and maintaining flap integrity are of greater clinical importance than surveillance alone.

Ischemic skin necrosis occurring immediately postoperatively or during its management (e.g., debridement) may disrupt the skin barrier and create a local environment favorable for bacterial colonization.

Although wound complications do not represent infection-specific perioperative risk factors, they reflect an unfavorable postoperative clinical course that may predispose patients to delayed infection during the prolonged tissue expander retention period. Accordingly, wound complications were interpreted as an associated postoperative condition rather than a direct causative factor of delayed SSI.

Consistent with this interpretation, Leyngold et al 34 reported that cellulitis, wound dehiscence, and breast flap necrosis were associated with an increased risk of infection during tissue expansion. Similarly, Kraenzlin et al 35 demonstrated that full-thickness skin necrosis, postoperative seroma, and prolonged drain duration were associated with expander infection on multivariable analysis.

Besides differences in risk factors, clinical features differed between the acute and delayed infection groups, which emphasized the importance of recognizing delayed infection. First, delayed infection significantly extended the periods from symptom onset to hospital visit. As several months have passed since their surgery, patients frequently feel that their surgical site is stable. Even when there are systemic symptoms, including fever, patients may not attribute the problem to the surgery. Moreover, the skin color or texture can change when patients receive radiotherapy, and signs of SSI, such as erythema, may not be noticeable. Furthermore, owing to the insensitivity of mastectomy flaps, infection frequently presents as pain and fever, typically when the infection is more severe. Second, delayed infection is more challenging to treat, increasing the possibility of explantation. A previous study by Franchelli et al 7 reported that implant salvage was possible in 41% of acute cases where infection developed <60 days postoperatively; in contrast, salvage was possible in only 12% of delayed infections. Sinha et al 3 observed a tissue expander explantation rate of 46% (30/65 cases) for delayed infections compared with the 27% (14/52 cases) in acute infections. In the present study, cases of acute infection demonstrated a salvage probability of 83% (5/6 breasts), whereas the rate of salvage in cases of delayed infection was low at 31% (4/13 cases). Of the nine breasts that could not be salvaged, two were reconstructed with an abdominal flap, and the remainder underwent abandoned reconstruction. Thus, breast reconstruction is significantly more likely to fail when delayed infection develops, reinforcing the need for surgeon and patient awareness. The low salvage rate can be associated with radiotherapy. 36 When an infection occurs in a radiated breast, the fibrotic and thinned skin flap is frequently in such poor condition that it cannot accommodate the new expander. Therefore, reconstruction using autologous tissue is usually necessary.

As with other retrospective studies, this study is subject to the potential for selection bias. However, a consistent definition of implant infection was applied, and only objective data extracted from medical records were used to minimize bias. In addition, the predominance of Asian patients reflects the local population demographics. Consequently, the inclusion of patients with relatively lower BMI and smaller breast weight compared with Western populations represents a limitation of this study.

The relatively small number of delayed SSI cases may have introduced bias and increased the risk of overfitting in the multivariable analyses, particularly for variables with strong collinearity.

Furthermore, final skin tension, which may affect tissue perfusion and wound healing, was not quantitatively evaluated. In routine clinical practice, skin tension during tissue expansion is assessed subjectively, and objective measurement is not feasible. This constitutes an inherent limitation of the present study.

### Conclusion

Delayed SSI following expander-based breast reconstruction is not rare and differs substantially from acute infection in both risk factors and prognosis. Because early diagnosis and salvage are challenging, surgeons should be alert to the risk of delayed infection, particularly in patients with wound complications that compromise postoperative wound integrity. Other clinical factors identified in univariable analyses may reflect patients at higher risk but should be interpreted with caution. Patients with these risk factors should be carefully informed to seek prompt medical attention when infection-related symptoms occur, so that timely treatment and possible expander salvage can be achieved.
